# Without birth, without death—issues in the research of nondual awareness or consciousness itself

**DOI:** 10.3389/fpsyg.2025.1665826

**Published:** 2025-11-25

**Authors:** Zoran Josipovic

**Affiliations:** Psychology Department, New York University, New York, NY, United States

**Keywords:** nondual awareness, non-duality, consciousness, reflexivity, awareness of awareness, meditation, mindfulness, minimal phenomenal experience

## Abstract

The drive to find one unifying theory, one overarching universal principle that would subsume and explain all, has been a common theme of human striving for knowledge for many centuries. It reflects, perhaps, a deep intuition that within every experience there is such a unifying singular essence, present yet ordinarily hidden, which when discovered would restore one to the wholeness of authentic being. Indeed, as contemplative traditions tell us, that singular essence is consciousness itself, that which is, and has been, aware or conscious in all our experiences. This nondual awareness, free from mental representations and their constructs, appears uncaused by anything other than itself and remains, once realized, relatively unchanged in different experiences. So, metaphorically, it is without birth or death. While the current increase in our understanding of consciousness has been, in many respects, exponential, our understanding of nondual awareness appears still elusive and more like a slowly ascending spiral, where each generation makes their unique contributions while still making largely the same mistakes. Like nondual awareness itself, then, many issues in understanding and researching it seem to be eternal as well. Previously, I have introduced nondual awareness and discussed why its non-representational reflexivity makes it unique and distinct from perceptual, affective, and cognitive contents, from various global states both natural and altered, and from functions of consciousness like attention, memory, meta-cognition, etc. Here, I will sketch out some issues related to ontology, epistemics, participant reports, and experiment design.

## Introduction

The drive to find one unifying theory, one overarching universal principle that would subsume and explain all, has been a common theme of human striving for knowledge for many centuries. It reflects, perhaps, a deep intuition that within every experience there is such a unifying singular essence, present yet ordinarily hidden, which, when discovered, would restore one to the wholeness of authentic being. Indeed, as contemplative traditions tell us, that singular essence is consciousness itself, that which is and has been aware or conscious in all our experiences. This nondual awareness, free from mental representations and their constructs, appears uncaused by anything other than itself and remains, once realized, relatively unchanged in different experiences. Thus, metaphorically, it is without birth or death. While the current increase in our understanding of consciousness has been, in many respects, exponential, our understanding of nondual awareness appears still elusive and more like a slowly ascending spiral, where each generation makes its unique contributions while still making largely the same mistakes. Like nondual awareness itself, then, many issues in understanding and researching it seem to be eternal as well.

Significant progress has been made in recent years in understanding human consciousness in terms of phenomenal content, functions that create and influence it, and global states of consciousness (Albantakis et al., 2023; Block, 2023; Chalmers, 2024; Cogitate Consortium et al., 2025; Deco et al., 2025; Fazekas et al., 2024; Koch, 2024; LeDoux, 2023; Mashour et al., 2020; Mudrik et al., 2025; Northoff and Ventura, 2025; Overgaard and Sandberg, 2021; Sandved-Smith et al., 2025; Seth and Bayne, 2022; Simione et al., 2025; Timmermann et al., 2025; Tononi et al., 2024).

However, consciousness itself, or consciousness as such, a.k.a. nondual awareness, is still insufficiently researched. Recently, promising advances have been made in this area (Agrawal and Laukkonen, 2024; Alcaraz-Sánchez, 2024; Atad et al., 2025; Boly et al., 2024; Lutz et al., 2025; Dunne et al., 2019; Metzinger, 2020, 2024; Sandved-Smith et al., 2025; Timmermann et al., 2025). However, these efforts can be plagued by certain misunderstandings about what nondual awareness is and how it is related to other aspects of experience.

I have previously introduced nondual awareness and discussed why its non-representational reflexivity makes it unique and distinct from other aspects of consciousness. Specifically, I outlined how it differs from both focused attention and open monitoring (Josipovic, 2010, 2013, 2014, 2019); from memory and metacognition (Josipovic, 2019, 2024); from perceptual, affective, and cognitive content, including a mere absence of content (Josipovic, 2019, 2021); from global states of consciousness, including altered states (Josipovic, 2019, 2021, 2025); and from the unconscious substrate (Josipovic, 2019, 2021). I have discussed non-representational reflexivity as a key property of nondual awareness, i.e., consciousness itself, in some detail (Josipovic, 2019, 2024). I will not repeat those points here, but most of them will become evident in the sections below. Additionally, I have previously contextualized this view of consciousness in relation to some of the main current theories of consciousness (Josipovic, 2019, 2021). Likewise, these will not be the topic of the present discussion. Here, I will outline some issues related to ontology, epistemics, participant reports, and experimental design.

The scope of issues in these areas is large, and some have been debated for centuries (Rabjam, 2007; Radhakrishnan, 1994; Mathes and Kemp, 2022). They cannot be fully addressed here. Hence, the style here will be that of an opinion paper, which points to issues rather than that of a review that thoroughly examines them. In keeping with that, the discussion is less of an analytical argument and more of an integrative description and pointing through metaphors, the essence of which can be seen as having some parallels to fundamental debates between analytic and continental philosophies (Tomasetta, 2024). However, these are not the topic here. The main approach used here could be characterized broadly as neurophenomenological (Berkovich-Ohana et al., 2020; Varela et al., 1993) and more precisely as nondual contemplative phenomenology. The term nondual contemplative here means that investigation is via nondual contemplation directed toward knowing through nondual awareness, or more accurately, via abiding in and as nondual awareness (Josipovic, 2019, 2024). At times, other methods are used, which can be seen as heuristic, hermeneutic, or even deconstructive. However, as often pointed out by nondual traditions, logical analysis alone cannot lead one to nondual awareness since nondual awareness is without mental representations (Gyamtso and Hookam, 2001; Higgins, 2013; Rabjam, 2007; Radhakrishnan, 1994). At best, it can lead to the exhaustion of conceptual grasping, and in doing so, create a space to experience nondual awareness directly (Lama, 2004). Thus, arguing about nondual awareness by showing inconsistencies and contradictions in statements about it, as if these were outcomes of logical analysis rather than descriptions of how it appears phenomenally, is not the most useful approach, as such arguments often end up being strawman arguments (Garfield, 2023). Insistence on refuting nondual awareness via logical arguments is also a reflection of the influence of natural direct realism and its implicit beliefs that conceptual structures accurately represent the phenomenal structures of experience, which can be true for conceptually constructed experience but is not the case for nondual awareness since it functions in a very different way from conceptual representational knowing.

The view of nondual awareness presented here is inspired by Asian nondual contemplative philosophies and traditions. While it is more similar to some than to others, it does not advocate for any specific doctrine. Likewise, there is no commitment to any metaphysical belief. The view presented here is based on current developments in consciousness research, on phenomenal reports of contemplatives both ancient and recent, and on my forty-plus years of experience with contemplative practices. As often pointed out (Dunne et al., 2019; Lutz et al., 2015), both traditional and contemporary accounts of nondual awareness, and of consciousness in general, can suffer from conflating phenomenal, epistemic, ontological, metaphysical, methodological, and soteriological levels of discourse. The present discussion will concern itself primarily with phenomenal and epistemic levels, and with subjective ontology. All descriptions and metaphors used here pertain to human experience only.

A note about the use of language is needed when dealing with the topic of nondual awareness and nonduality. Language is linear, dualistic, and temporal, with a subject and object structure, while nondual awareness is nondual, holistic, and atemporal, so discourse on this topic can easily appear circular and paradoxical, like attempting to describe a three-dimensional sphere using a one-dimensional line (Josipovic, 2016, 2021). Language demands that a statement be expressed as, “one experiences pure nondual awareness as…,” but during such events, there is no separate subject experiencing nondual awareness as if it were an object; there is only nondual awareness that knows itself inherently. Paradoxical statements, such as nondual awareness being simultaneously transcendent and immanent in experience, or the well-known one, everything is already perfect just as it is, are descriptions of how nondual awareness appears, and, respectively, how phenomena appear when they are co-present with it. Conversely, because nondual awareness is a different way of knowing from the one based on mental representations, statements that seem valid and reasonable when applied to everyday experience and consciousness do not necessarily apply to nondual awareness (Josipovic, 2021).

Terminology has been a perennial issue in consciousness studies (Cardeña et al., 2025; Van Gulick, 2025). Over the centuries, different contemplative traditions have developed their own unique terminologies, frequently using different terms for the same aspects of consciousness (Rabjam, 2007; Radhakrishnan, 1994). In respect to contemporary consciousness research, terms from contemplative traditions often have different meanings from how they are used in cognitive science, neuroscience, and philosophy (Dunne et al., 2019; Lutz et al., 2025; Josipovic, 2019, 2024). A few key terms are therefore defined below.

- *Consciousness, in general*, refers to experience with all its aspects (Van Gulick, 2025). These include phenomenal content, functions of consciousness which are processes that create and influence content, global state, indeterminate substrate or the unconscious, and consciousness itself or nondual awareness (see below for details; Josipovic, 2019).
- *Phenomenal content* refers to perceptual, affective, or cognitive, content of experience (Block, 2023; Seth and Bayne, 2022).
- *Functions of consciousness* refers here to processes like attention, memory, decision-making, imagination, meta-cognition, etc., which are involved in creating and influencing phenomenal content. To the extent that these processes are conscious in an experience, they are aspects of that experience. This term is to be disambiguated from other uses of the term function, for example, an evolutionary function of consciousness (Cleeremans and Tallon-Baudry, 2022; Fleming and Michel, 2025; Ludwig, 2022, 2025) or a function of nondual awareness in experience (see further below).
- *Global state* refers to the overall state one is in, which can be naturally occurring, such as waking, dreaming, or deep sleep, or induced, such as a psychedelic altered state, contemplative state, or minimally conscious state (Bayne et al., 2016; Seth and Bayne, 2022).
- *Indeterminate substrate* refers here to the underlying conceptual structures which, together with memory traces, shape the experience. The substrate is neither fully conscious nor entirely non-conscious. It is similar to the psychoanalytic cognitive unconscious. The indeterminate substrate functions as a pervasive potential or matrix for structuring experience (Germano and Waldron, 2006). When nondual awareness fails to recognize itself, when its non-representational reflexivity is not activated, the substrate functions as dualistic subject-object structuring through which consciousness itself manifests as a tripartite representational process of knower-knowing-known (Josipovic, 2014, 2019, 2021).
- *Non-dual awareness* refers to consciousness itself or consciousness as such, a foundational awareness that does not rely on mental representations to know, and so it does not structure experience into the conceptually reified duality of subject and object (Higgins, 2013; Josipovic, 2014, 2019, 2021, 2024; Lingpa and Wallace, 2017; Metzinger, 2020, 2024; Norbu, 2013; Rabjam, 2001; Wilkinson, 2018). The term nondual awareness is used here irrespective of the type and amount of other phenomenal content co-present with it or the specifics of the global state. Awareness here always means conscious awareness, whether it is non-representational or representational. Non-conscious awareness is not awareness here, but unconscious detecting. Related terms, pure awareness or pure consciousness, refer to nondual awareness when it is present without other phenomenal content, as can occur in minimal phenomenal experience (see Metzinger, 2020, 2024; for disambiguation see Josipovic, 2019). There are numerous traditional and contemporary terms, such as clear light awareness, timeless awareness, the Self (with a capital “S” for disambiguation), choiceless or non-propositional awareness, self-knowing awareness, etc., which refer to the same nondual awareness.
- *Reflexivity* refers here only to awareness of being aware, in other words, to consciousness knowing itself to be conscious. Reflexivity can be indirect via mental representations or direct non-representational (Gallagher and Zahavi, 2023; Garfield, 2006; Josipovic, 2019, 2024; Montague, 2016; Zahavi, 2005). The term, as used here, does not refer to reflecting on and introspecting one's experience. It does not have any other meaning either, such as those used in social research, etc.
- *Non-dual reflexivity* refers to direct, non-representational knowing by which nondual awareness knows that it is aware. It is an intrinsic property of this awareness rather than an intentional transitive act. Hence, adjectives inherent or intrinsic apply to this reflexivity (for a detailed discussion see Josipovic, 2024; see also Gyamtso and Hookam, 2001; Higgins, 2013; Williams, 2000). This differentiates nondual reflexivity from both reflective and pre-reflective self-awareness as these terms are commonly used, since they are based on mental representations.
- *Realization* refers to the activation of this nondual reflexivity (Norbu, 2013; Rabjam, 2001). By extension, it can refer to knowing oneself as, or abiding as, nondual awareness (Radhakrishnan, 1994). It does not mean to understand something conceptually or to have an aha moment, as the term is usually used. Related terms, self-recognition and self-knowing, refer to the same. Non-representational reflexivity has also been termed self-knowing, and its activation a self-recognition (Ksemaraja and Singh, 1990; Rabjam, 2007). Such awareness is then termed self-knowing awareness or self-awareness for short (Higgins, 2013). These terms do not refer here to their usual meanings in psychology and cognitive science of recognizing or knowing one's constructed self.

- *Non-duality* refers to how experience appears in nondual awareness, as nondual, without subject-object conceptual structuring. Conversely, duality is how experience ordinarily appears, as conceptually structured into subject-object polarity. On a deeper level, nonduality refers to the simultaneous transcendence and immanence of nondual awareness, meaning that nondual awareness, when explicitly present in an experience, is simultaneously transcendent and immanent to phenomena (Josipovic, 2014, 2021; Rabjam, 2001; see further below for details). Other meanings of the term nonduality, such as an absence of self-related content, an absence of environment-related content, or an absence of both, as in unconscious absorption, are here regarded as only partially nondual because they entail rejecting aspects of experience and thus creating another, albeit more subtle, duality.
- *Representation* refers here to mental representations only, such as concepts, schemas, propositions, and semantic, iconic, or numeric symbols (Shea, 2018). It does not refer to neural or any other representation. Conceptual categorization refers to categorization by labeling, as opposed to non-conceptual categorization by segregation or exclusion (Lamme, 2020; Thompson, 2021). On the view presented here, both non-representational and representational knowing are not only possible but necessary.
- *Non-representational* here means without mental representations.
- *Conceptual reification* refers to a process of conceptually representing experience and solidifying those representations into subject and its objects that appear as separate and fundamentally different (Lutz et al., 2015; Rabjam, 2007).
- *De-reification* refers to the opposite of the above, i.e., seeing through and disrupting these reification processes (Dunne et al., 2019; Lutz et al., 2025). A related term is derealization, which refers to seeing through the implicit belief that objective reality is exactly as it appears in one's conceptually reified experience. This has a different meaning from psychiatric derealization, which is a loss of orientation to time, space, etc.

I will next summarize the key features of nondual awareness that are relevant for the discussion of issues below. Non-dual awareness is a type of awareness that knows both itself and phenomena without relying on mental representations and without structuring experience into the conceptually reified duality of subject vs. object (Dunne et al., 2019; Higgins, 2013; Josipovic, 2014, 2019, 2021, 2024, 2025; Lingpa and Wallace, 2017; Rabjam, 2001, 2007; Wilkinson, 2018). It is a different kind of knowing from the one we usually rely on, which requires mental representations (Shea, 2018). In that sense, it is different from both fast and slow thinking, and from both reflective and pre-reflective consciousness that rely on mental representations (Gallagher and Zahavi, 2023; Kahneman, 2011; but see Williford et al., 2022 for non-representational pre-reflective awareness).

Phenomenally, nondual awareness appears as entirely empty, silent, unmoving, and clear, like empty space that is luminously aware but does not do anything (Josipovic, 2014, 2019, 2021, 2024; Lama, 2004; Lingpa and Wallace, 2017; Metzinger, 2024; Rabjam, 2001, 2007; Radhakrishnan, 1994; Wallace, 2024; Wilkinson, 2018). Functionally, it occurs after some minimal level of arousal or alertness is present, but before any attentional selection, either involuntary or voluntary. Its function is to simply mirror whatever content and state is present in experience, including its own presence (Rabjam, 2001; Norbu, 2013). This basic mirroring function occurs most likely via some spontaneous recursive resonance, or mirroring resonance for short (Josipovic, 2021, 2024; Laukkonen et al., 2025; Sandved-Smith et al., 2025). It can be compared to a mirror merely reflecting whatever is present in front of it (Norbu, 2013). This mirroring precedes mental representations, so it is before categorization, labeling, associating, evaluating, or decision-making. It is before the most rudimentary innate conceptual structures such as inside vs. outside and subject vs. object, which constitute the unconscious substrate (Germano and Waldron, 2006; Higgins, 2013; Mathes and Kemp, 2022). It occurs before any interpretation of sensation-perception and before any response or action, from basic appetitive response to complex contextually directed action. So it is before language, imagination, reasoning, and meta-cognition. However, the designations “before” and “after” as applied here do not necessarily indicate temporal sequence but instead point to the degree of functional complexity. Non-dual non-representational awareness is the simplest unconstructed awareness that is conscious and that can know that it is conscious directly or non-transitively. In that sense, it is consciousness itself or consciousness as such (Gyamtso and Hookam, 2001; Josipovic, 2019, 2021, 2024, 2025; Rabjam, 2007; Radhakrishnan, 1994).

Non-dual awareness is thought to be present in every conscious experience, though usually only implicitly, covered up by habitual conceptualizations about subject and object. It can become explicit and recognize itself; in other words, its reflexivity can become activated. When nondual awareness is implicit, we know that we are aware only indirectly via same-order or higher-order representations (Brown et al., 2019; Josipovic, 2024; Montague, 2016; Kriegel, 2024; Rosenthal, 2012). The direct non-representational reflexivity of nondual awareness is then obscured. When it is not obscured, nondual awareness knows that it is aware directly or non-transitively, without mediation by mental representations, as its intrinsic property. This type of reflexivity can be termed nondual reflexivity. It is unique to nondual awareness and makes it what it is (for detailed discussion, see Josipovic, 2024; Williams, 2000). Non-dual awareness does not define or specify itself in any way, as it is self-evident to itself (Gyamtso and Hookam, 2001; Josipovic, 2019, 2024; Rabjam, 2007).

The type of knowing also influences how phenomenal content appears. Properties of knowing become reflected, so to speak, in phenomena, appearing as more or less universal properties of phenomena. For example, knowing via opposing conceptual representations of subject and object makes experience appear as intrinsically fragmented and structured into opposing dualities of self vs. other, good vs. bad, us vs. them, etc. On the other hand, knowing via nondual awareness reveals emptiness, being, luminosity, bliss, and unity or nonduality as their universal properties (see below for details: Josipovic, 2021, 2025; Rabjam, 2001). This can be understood as a change in the implicit-explicit gradient of nondual awareness, from ordinary dualistic experience in which nondual awareness is entirely implicit to fully nondual, in which it is explicit and its nondual reflexivity is unobscured (for detailed discussion, see Josipovic, 2021).

The more explicit nondual awareness is in an experience, the more its dimensions are mirrored in perceptions, affects, and cognitions. When nondual awareness is fully explicit, experience appears as nondual or “pure,” and any phenomena unfolding within its space are “sealed” with its dimensions or properties (Rabjam, 2007; Josipovic, 2014, 2021, 2024). Non-dual awareness is then experienced as simultaneously transcendent and immanent in conscious contents and states. It is transcendent as the silent aware space that pervades and encompasses the entire conscious experience, and it is immanent as that out of which everything is made, the way water in a glass is both the medium in which ice cubes float and the substance out of which they are made (Josipovic, 2016, 2021, 2024, 2025).

Once explicit, nondual awareness appears phenomenally as singular and homogeneous. Nevertheless, it has distinct qualities or dimensions that are its essential properties. These are not dependent on being conceptualized but are spontaneously self-evident as what nondual awareness is. Importantly, the properties of nondual awareness are not separate elements from which this awareness is assembled or from whose interactions it emerges (Deutsch, 1980; Josipovic, 2019, 2021; Laish, 2015; Metzinger, 2020, 2024; Rabjam, 2007).

A number of different dimensions and qualities can be found in contemporary and traditional reports (Rabjam, 2007; Higgins, 2013; Josipovic, 2019, 2021; Metzinger, 2024; Fasching, 2021). Below is a brief summary of those that are here regarded as intrinsic:

- *Being or presence*—the obvious fact of awareness being present or phenomenally existing.
- *Emptiness—*an absence of conceptually assigned identity and conceptualizations about itself or phenomena that reify awareness as the subject and phenomena as objects.
- *Non-dual reflexivity*—nondual awareness inherently knowing itself to be aware without relying on mediation by mental representations; also, non-transitive reflexivity.
- *Luminosity—*the quality of cognitive reflexive knowing of nondual awareness. This luminosity is not something awareness knows as perceptual content, but what awareness itself is.
- *Bliss*—the silent contentment of being entirely complete in itself, with no sense of any lack or any need for anything outside of itself; so in this sense, without intention.
- *Singularity or unity*—nondual awareness is singular and homogenous, a unity of all its dimensions, not compounded or constructed from them or from anything else.

The above dimensions or properties are intrinsic and present regardless of whether nondual awareness is co-occurring with other phenomenal content or entirely without it. Other closely related dimensions, which can be regarded as corollaries of the above ones, are boundless spaciousness, non-locality, timelessness, and self-sameness.

The boundlessness of space means that the space of nondual awareness is singular and homogenous, and it encompasses and pervades all contents within one's perceptual bubble, in both internal and external environments, without conceptually constructed boundaries (Blackstone, 2007; Josipovic, 2014, 2021; Metzinger, 2024; Rabjam, 2001). It is phenomenally infinite because no end edge can be detected in it. Non-local here refers to the intrinsic properties of nondual awareness pervading the entire space of experience equally. Self-same means that nondual awareness and its intrinsic properties remain unchanged in different experiences, irrespective of the changing phenomenal content or global state. While spaciousness is a property of nondual awareness, and nondual awareness, when explicit, functions as the epistemic space of experience (Metzinger, 2020; Josipovic, 2014, 2019; Rabjam, 2001), it would be a mistake to see nondual awareness as a derivative of that space or of its representations (Metzinger, 2024; Lee, 2024; Laukkonen et al., 2025). Rather, nondual awareness, consciousness as such, is a unique kind (Josipovic, 2019, 2025).

Non-dual awareness also appears to be atemporal, without past or future as they are ordinarily experienced (Laukkonen and Slagter, 2021; Metzinger, 2024; Sandved-Smith et al., 2025). Since it is without mental representations, nondual awareness does not have beliefs about the past or predictions about the future. In itself, it is capable of only relatively short, non-representational, resonance-sustained mirroring memory, which is experienced as ongoing nowness (Block, 2023; Josipovic, 2019, 2021, 2025). However, the nowness of nondual awareness is not a present moment sandwiched, as it were, between moments of past and future. Such experiencing of time is indicative of the presence of an implicit subject as attender who is observing a succession of moments, all of which are absent in nondual awareness. Similarly, the belief that there is no present because any observed moment is either already in the past or has not yet occurred indicates that one has not yet arrived at nondual awareness and is still involved with observing the temporal sequence. Non-dual awareness is, so to speak, before working memory, monitoring, and representational knowing that enable notions of past and future (Josipovic, 2019, 2025).

Further dimensions of nondual awareness that are often reported but are not intrinsic to it appear as qualities of being when explicit nondual awareness is embodied—in other words, when it is reflected in somatic, affective, and cognitive processes. For example, qualities of strength, happiness, love, and compassion appear then as spontaneous qualities of one's authentic being, rather than as outcomes of transactions (Josipovic, 2016). Other indirect qualities may manifest as ethical ideals in relational and social contexts.

The issue of the relationship between nondual awareness and the self is complex and has been much debated (Blackstone, 2007; Berkovich-Ohana et al., 2024; Coseru, 2024; Garfield, 2020, 2023; Metzinger, 2024; Rabjam, 2007; Thompson, 2017; Zahavi, 2025). It is outside the scope of this paper but needs a summary here. Current research indicates that the self is a multilayered construct consisting of a number of processes that form layers or patterns. A well-known map used in neurosciences and consciousness research groups these into three (Gallagher et al., 2024; LeDoux, 2023). The first, proto-self, relates to largely unconscious homeostatic and allostatic mechanisms manifesting as interoceptive signals; the second, core or minimal phenomenal self, comprises body ownership, spatial perspective, and agency; and the third, extended self, is made up of processes involved in autobiographical, narrative, executive, and various social identities. Other maps refer to two kinds of self; for example, dual process low self and high self, where the low self may be comprised of proto and core self as the minimal phenomenal “I,” and the higher extended self as the reflective access “me” (Dahl et al., 2015). Alternatively, the false self and the true self relate to the degree of psychological authenticity (Winnicott, 1965). To the extent that these layers or patterns depend on mental representations, they are constructed selves. In line with some contemplative traditions, a fourth type of self is sometimes posited as related to pure consciousness itself, i.e., nondual awareness, which is not constructed in the same way as other selves. Alternatively, an abstract concept of a transcendent self is posited for this fourth self, which, however, would still be a mental representation irrespective of how abstract it is (Garfield, 2023; Blackstone, 2007; Rabjam, 2007; Radhakrishnan, 1994).

Different layers of the constructed self can be temporarily suspended from experience via meditation, psychedelics, or spontaneously (Berkovich-Ohana et al., 2024; Chiarella et al., 2024; Fingelkurts et al., 2020; Sparby and Sacchett, 2024). However, several mistaken inferences about the nature of the self can occur based on these experiences. The fact that this self is constructed does not mean that it does not exist; just that it is not a monolithic, unchanging entity we usually implicitly assume it to be. A related erroneous belief is that the constructed self must be eradicated in order to attain nondual awareness (Albahiri, 2024). This belief is based on a misunderstanding that creates yet another duality, one in which self-related content is not allowed in consciousness. It also represents a lack of deeper understanding that, however paradoxically, the fundamental wholeness of experience includes both nondual and dual experiencing (Josipovic, 2021, 2024, 2025; Rabjam, 2007).

What is important to understand for our discussion is that constructed layers of self, whether proto, core, or extended, are not intrinsic to nondual awareness. In that sense, nondual awareness is without a self. In another related sense, as we will see further below, it is also without a self as it does not reify itself conceptually; it is not a transcended conceptual self. Yet, phenomenally, once it is clearly explicit, nondual awareness is the most essential self. It is who or what is conscious or aware, non-representationally, in any experience. However, while distinct from them, this awareness is not a self that is separate from experience; it is not separate from any content and state that is co-present with it; rather, it is nondual with it (Blackstone, 2021; Josipovic, 2021, 2025).

Furthermore, since nondual awareness has no preference for what content or state unfolds within it, it is not the self as the one who is attending, monitoring, recollecting, planning, deciding, and controlling, etc., prompted by conscious and unconscious motivations (Josipovic, 2024, 2025). Non-dual awareness is not a property or a capacity of any constructed self, nor of any of its layers and processes. It is also not a property of some abstract transcendent self, no matter how far outside of experience and consciousness we may imagine it to be (Rabjam, 2007; Garfield, 2023). Rather, to the extent that any such conceptually based self is consciously present, it is a content occurring within nondual awareness (Josipovic, 2019, 2021, 2024, 2025).

Barring some dissociative patterns, reports that nondual awareness appears not to be one's own experience often point to the fact that it is not a capacity or experience belonging to one's constructed self. These reports can also be indicators of relatively early stages of discovery of nondual awareness when one is still strongly identified with one's constructed self (Metzinger, 2024). The self-recognition of nondual awareness by itself is then still on and off, and this awareness has not yet become stable enough for one to realize that it is not anything other than who one essentially is as conscious presence. Even though it is, in itself, entirely empty of the usual layers of self, nondual awareness is not something alien or other. Rather, it is who one most intimately is. So, even in instances of completely pure nondual awareness without any other phenomenal content and without any layers of constructed self, it is never a question of whether it is you who is aware, or maybe it is your cousin (Josipovic, 2019, 2021).

## Existence issues

The ontological status of consciousness has been a perennial question since the dawn of human civilization (Kuhn, 2024). Here, we are concerned with consciousness as such or nondual awareness only as an aspect of human experience. As such, nondual awareness is subjectively real (Josipovic, 2025). The question of whether nondual awareness, or for that matter consciousness in general, has any objectively real status independent from the human brain is outside the scope of this discussion. Even though nondual awareness is without conceptually constructed subject and object, and may appear to be phenomenally more real than the ordinary conceptually constructed experience (Metzinger, 2024), this does not mean that the subjectively real and the objectively real are the same (Searle, 2017). The two are distinct but do not negate one another. For example, when nondual awareness is explicit and immanent in experience, everything can appear as if it were made from the same awareness, including, for example, the floor one may be standing on. However, if one were to fall, the objective reality of the floor would become painfully obvious.

Non-dual awareness can also become reified into conceptual beliefs about the metaphysical status of consciousness. For example, everything we experience is our consciousness, by definition. After realizing nondual awareness, one can experience that, from its side, everything appears as one infinite consciousness. But to then assume that indeed there is one consciousness underlying and subsuming the entire universe and everything in it—in other words, that this is also objectively real—constitutes a leap of inference (Faggin, 2023; Kastrup, 2024). Conversely, an a priori rejection of the possibility of this universal consciousness would similarly be a mistake. Thus, an overall epistemic stance of caution is needed when dealing with this topic.

Perhaps the most commonly found obstacle to advancing research on nondual awareness is the belief that it does not exist (Josipovic, 2024; Metzinger, 2024). Besides the obvious reason of not wanting to waste time chasing non-existents, the problem with this belief is that without understanding that nondual awareness is phenomenally real and unique, a mistaken inference can be made that it is merely a result of conceptually mislabeling some other aspect of experience, like wakefulness, attention, monitoring, or meta-cognition (Dunne et al., 2019; Josipovic, 2019; Metzinger, 2020).

One of the arguments for the non-existence of nondual awareness centers on the claim that all phenomena are impermanent and therefore do not exist in themselves; likewise, nondual awareness does not exist in itself (Garfield, 2023; Gyamtso and Hookam, 2001; Mathes and Kemp, 2022). To begin with, nondual awareness is not a phenomenon; rather, as consciousness itself, it is the only true noumenon available to us (Josipovic, 2024; Williams, 2000). More to the point, nondual awareness is not impermanent in the way that perceptual, affective, and cognitive contents are. As the background awareness in all conscious experiences, nondual awareness remains relatively unchanging. To the extent that it is explicitly present or realized, it is relatively invariant in experience, in the sense that its intrinsic properties or dimensions remain the same (Josipovic, 2019, 2021; Rabjam, 2001, 2007; Radhakrishnan, 1994). The debate over its impermanence is sometimes presented as the issue of whether nondual awareness is continuous or discrete, i.e., whether it flickers on and off at some frequency, the way perceptual content is thought to be bound into frames by alpha waves, only faster or slower (Riddle and Schooler, 2024). Either way, this issue does not affect its phenomenal existence.

A related argument sees all conscious experiencing as reducible to perception, to a stream of ever-changing moments of perceiving and perceived (Kuhn, 2024; Rahula, 1994). However, this is not an accurate view with respect to nondual awareness. Non-dual awareness is orthogonal to perception, as it can be explicitly present independent of the amount or type of perceptual content, both without any perceptual content or with full perceptual content, as in normal wakefulness (Josipovic, 2014, 2019; Metzinger, 2024).

The fact that nondual awareness is usually only implicit and not discoverable through the usual conceptual methods such as introspection is not proof that it does not exist but evidence that introspection and other conceptually based methods are not adequate for arriving at non-conceptual awareness. Furthermore, nondual awareness does not have the dualistic structure of conceptual cognitions (Sansk. vijnana). Even the basic splitting of experience into perceiving and perceived is absent in it, along with all subsequent conceptualizations and post-perceptual inferences (Gyamtso and Hookam, 2001; Mathes and Kemp, 2022; Rabjam, 2007). Thus, nondual awareness is not a series of moments of perception and perceptual knowing that arises in response to sensory inputs. Rather, it is a steady, all-encompassing aware space within which perceptions appear and disappear (Rabjam, 2001; Josipovic, 2014, 2021, 2024; Metzinger, 2024). The claim that any presence of nondual awareness in experience is evidence that one is still engaged in perceiving and that awareness is an object of that perception (Rabjam, 2007) is based on a misunderstanding that nondual awareness knowing itself is not a transitive subject-knowing-object process but instead a property of awareness itself, so it can't be any kind of perception, no matter how subtle. Rather, this is a sign that nondual awareness is still known only indirectly as a conceptually reified subtle mental object (Josipovic, 2019, 2024). When nondual awareness is explicit and one abides nondually, there are no moments of perceiving and perceived. As nondual awareness is atemporal, everything that occurs in its epistemic space is present a-temporally, as nowness in which everything that is experienced, including perceptions, is one singular presence (Josipovic, 2024, 2025).

Another claim for the non-existence of nondual awareness is based on the partial understanding of the Buddhist teachings on the 12 links of dependent origination (Garfield, 2023; Rabjam, 2007). According to this interpretation, everything one experiences is created through causal chains of dependent origination and is empty of intrinsic existence, including nondual awareness. However, such dependent origination applies only to conditioned phenomena that are constructed through sequences of cause-and-effect relations originating with ignorance. Ignorance here is a non-recognition of awareness by itself, an impediment to its reflexivity, which leads to replacing awareness with a conceptualized subject and phenomena with conceptualized objects, which are then further reified, resulting in the progressive cognitive and affective fragmenting of experience (Guenther, 1984; Metzinger, 2024; Rabjam, 2007). Since nondual awareness is without such ignorance and is a primordial non-conceptual knowing (Sansk. prajnana; Tib. yeshe), it is outside of such chains of cause and effect (Higgins, 2013; Josipovic, 2014, 2021, 2024; Rabjam, 2007). Therefore, nondual awareness is not dependently arising or empty in that sense. Phenomenally, the continuum of nondual awareness is its own cause (Lama, 2004). This is the meaning of the term self-originated when applied to nondual awareness (Higgins, 2013; Manjusrimitra and Lipman, 2001; Rabjam, 1998).

A contemporary reinterpretation of the above idea, influenced by relational structuralism and process metaphysics, is that nondual awareness does not exist in itself because it is interdependent, produced as an emergent property of interactions of its elements, or as an outcome of the activity of other cognitive functions (Garfield, 2023; Metzinger, 2024; Segall, 2025; Thompson, 2017). However, nondual awareness cannot be specified as an interaction of its elements because it is not made from parts. Its dimensions or qualities are not separate elements it is composed of, or from whose interactions it emerges, but are properties of one singular awareness that is uncompounded and homogeneous (Josipovic, 2019, 2021; Lama, 2004; Lama and Hopkins, 2010). Non-dual awareness is not constructed the way ordinary experience, like perception, is—through a series of steps and predictions, such as identifying, labeling, associating, evaluating, deciding, etc. (Lamme, 2020). Rather, it is an all-at-once presence, or, as the traditional metaphor states, fully grown from birth (Rabjam, 2001, 2007). Similarly, it is a unique kind and not produced as an output of the activity of other cognitive functions like attention, vigilance, monitoring, meta-cognition, memory, etc. (Rabjam, 2001; Josipovic, 2019, 2025; Manjusrimitra and Lipman, 2001). For example, while the progression of being aware of perceptual stimuli, attentional placement, monitoring, and meta-cognition or meta-awareness describes the progress of mindfulness meditation, which on occasion may end in an isolated nondual awareness whose reflexivity is activated (Sandved-Smith et al., 2025; Metzinger, 2024), it would be a mistake to think that nondual awareness is a product of such a chain of cognitive and meta-cognitive processes (Josipovic, 2024, 2025).

It can also be argued that nondual awareness depends on neural processes in the brain and that, therefore, it does not exist as such. Accepting the current understanding that all aspects of conscious experience, including nondual awareness, depend on some neural activity, this is still not a reason to think that nondual awareness does not exist phenomenally. The point here is not whether nondual awareness is objectively real or whether it can exist independently of the human brain, but that it is phenomenally unique and irreducible. A further argument can be made in line with enactivist views, according to which conscious experiencing is a product of the brain's and body's interdependence with the environment and of an agent's interactions with the environment (Thompson, 2017; Varela et al., 1993). While on a biological level this view makes sense in terms of the survival of an organism, phenomenally, it does not apply to nondual awareness, which is, as mentioned above, outside the perception-action cycle. Non-dual awareness does not depend on the presence or absence of phenomenal content, whether that content is related to body interoception or environment exteroception. Likewise, it does not depend on any action taken in response to those or as an interaction with them (Josipovic, 2021, 2025).

Beliefs in the non-existence of nondual awareness also come from the commitment to literalist interpretations of dependent origination and interdependence, according to which the only good self is no self, and the only good consciousness or awareness is no consciousness or awareness at all. According to these views, all experiencing must cease to arrive at that which is real or true (Burbea, 2014; Prest and Berryman, 2024; but see also Sparby and Sacchett, 2024). In other words, only a complete absence of consciousness and experiencing of any kind, as in a cessation arrived at through meditative absorption (Sansk. nirodha, nirodha samapatti), is ultimately real. However, nondual awareness is not a state that is created through contemplative practice, the way various meditative absorptions, including cessation, are, and so it cannot be deconstructed in this way (Manjusrimitra and Lipman, 2001; Gyamtso and Hookam, 2001; Josipovic, 2025). It can be discovered by applying certain contemplative techniques, but it is not produced by them or dependent on them. Upon discovering it, one realizes that it is, and has always been, present in any conscious experience, only obscured by habits of conceptual mentation (Higgins, 2013; Josipovic, 2021, 2025; Mathes and Kemp, 2022; Rabjam, 2001; Wallace, 2024; Wilkinson, 2018).

Inferences about the non-existence of nondual awareness find support in those views that see all aspects of experience as conceptually constructed or as requiring access via mental representations to be conscious or aware (Brown et al., 2019; Gyamtso and Hookam, 2001; Lama, 2004; Rabjam, 2007; Rosenthal, 2012). Hence, it is believed that anything that is non-conceptual or non-representational cannot be aware or conscious (Siewert, 2022). The unconscious conceptual reifications make different aspects of experience appear as separate elements with fixed identities. These can be found at the level of relatively superficial subconscious narrative or at deeper levels of the self-world model. They also include foundational dualistic conceptual structures that underlie all ordinary experiencing (Berkovich-Ohana et al., 2024; Josipovic, 2021, 2024; Metzinger, 2010). Once this structuring of experience is understood, and especially once it has been consciously experienced, as can occur in contemplative practice, one can easily conclude that phenomena are merely conceptual designations and that, therefore, nothing exists in itself or as such (Garfield, 2023; Gyamtso and Hookam, 2001). A valuable aspect of this understanding is that nondual awareness is not a thing that is somehow separate from experience (Garfield, 2020; Higgins, 2013; Josipovic, 2021, 2025). If it appears as such, this is an indication that it has been replaced by a reified concept about it (Metzinger, 2024). But this does not mean that nondual awareness does not exist phenomenally or that it is not unique. Such belief in the non-existence of nondual awareness overestimates the importance of conceptualizations and conceptual reifications. Their involvement in constructing ordinary experience can significantly influence how we experience ourselves and our environment and can, at times, create an altogether imaginary experience. But this does not mean that an authentic phenomenal experience is not possible. For example, the temporary cessation of our conceptually constructed self-world model can appear at first as the often-reported dramatic end of our self and of the world as we know it, but only so because of the strength of our unconscious identification with our model and the degree to which we have replaced phenomenal experiencing with beliefs and predictions about it (Metzinger, 2024; Josipovic, 2016, 2025; Trautwein et al., 2024). Their absence then is not a mere nothingness, but a space that can reveal our authentic being (Blackstone, 2007; Josipovic, 2016; Laish, 2015; Klein and Wangyal, 2006). Similarly, when conceptual reifications related to nondual awareness and its qualities cease, it would be a mistake to conclude that nondual awareness or its qualities do not exist. Rather, it is then that they can become self-evident most easily (Josipovic, 2024, 2025). To a conceptual mind analyzing it, nondual awareness can appear as a mere negation, as not existing. But to itself, it is vividly present awareness with all its qualities (Gyamtso and Hookam, 2001; Josipovic, 2014, 2021, 2024).

Additionally, the above mentioned views correctly point to untenability of inferences about metaphysical status of consciousness based on phenomenality of awareness (Garfield, 2020; Higgins, 2013; Josipovic, 2025; Rabjam, 2007). But they also overextend this insight into equally metaphysical belief about its non-existence, and about non-existence of its qualities or properties. A valid question here is which of the qualities ascribed to nondual awareness are its intrinsic properties, and which are present only due to its being embodied or due to relational, social, and cultural contexts one is in. Wholesale rejection of qualities is usually based on a mistaken idea that they are present only because they have been conceptually reified, or culturally constructed (Garfield, 2023; Josipovic, 2025). Or, one can be experiencing them as separate elements, which is usually a sign that one has not yet realized them in themselves, but is instead experiencing their reflections, so to speak, in states of mind and body (Hanley et al., 2025). However, intrinsic qualities of nondual awareness are its essential properties that make it what it is, and are self-evident whenever nondual awareness is explicitly present, or self-recognized (Josipovic, 2014, 2019, 2021). So, it can be said that they exist primordially, unconstructed and beyond such division, origination, and cessation (Rabjam, 2001; Josipovic, 2021, 2025).

Non-dual awareness cannot be bound by mental representations and symbols about it, but that does not mean that nothing can be said about it. Arguments against the possibility of making any positive statements about consciousness itself or nondual awareness on these grounds, are comparable to arguments about properties of different fingers pointing at the moon, but are not about the moon itself (Duckworth, 2024). To a conceptual mind this awareness may seem ineffable, but to itself, it is entirely self-evident. This is due to the intrinsicality of its dimensions, and especially that of its nondual reflexivity (Josipovic, 2024).

Another related belief is that nondual awareness does not exist because it is an illusory appearance only. On this view, all experiencing is an illusory appearance, like a dream or a mirage; therefore, nondual awareness is likewise only an illusory appearance (Longchenpa and Guenther, 1975; Gyamtso and Hookam, 2001; Seth, 2021). This is thought to be similar to how the events and the protagonist in a vivid dream appear real, while both are a creation of the dreaming mind. Likewise, the objective and subjective aspects of waking experience appear real but are both created and influenced by the unconscious substrate, its conceptual structures, memory traces, beliefs, and future predictions (Germano and Waldron, 2006). On the view presented here, such claims of the illusoriness of nondual awareness are based on a mistaken conflation of nondual awareness with the unconscious substrate. Non-dual awareness is then seen as either a product that arises from the substrate or as the substrate itself that is revealed once its conceptual structuring and memory traces have been quieted down or purified (Mathes and Kemp, 2022). However, the fact that many cognitive processes are influenced by the unconscious does not necessitate that nondual awareness is produced or influenced in the same way. This is because nondual awareness is a knowing of a fundamentally different kind from processes based on mental representations and on dualistic conceptual structures of the substrate unconscious (Josipovic, 2019, 2024; Williams, 2000; Higgins, 2013).

This contributes to another mistaken idea: that nondual awareness is indeterminate (Higgins, 2013; Rabjam, 2007; Germano and Waldron, 2006). The substrate is phenomenally an indeterminate potential in that it is neither conscious nor non-conscious but can, in the presence of the right conditions, give rise to any conditioned experience. All such experiences, whether ordinary or altered, have the substrate's dualistic conceptual structures of subject-object, inside-outside, etc., as their implicit structuring. However, nondual awareness is in itself reflexively aware, non-representational, and without such conceptual structuring; hence, it is not indeterminate like the substrate (Gyamtso and Hookam, 2001; Josipovic, 2019, 2021, 2024, 2025; Mathes and Kemp, 2022). Non-dual awareness is also not created by purifying the substrate of dualistic conceptual structures, memories, and habit responses. This can be compared to how, when clouds part and reveal the sun, we don't say that the sun was produced by clouds. Again, this is because nondual awareness is a fundamentally different kind of knowing from representational conceptual knowing. In this sense, it is not illusory. Rather, it is that which is phenomenally real, beyond the constructed subject and object, whether in waking, dreaming, or deep sleep (Higgins, 2013; Josipovic, 2021, 2024, 2025).

Finally, perhaps the most subtle misconception regarding the non-existence of nondual awareness is that nondual awareness appears to exist only because it turns toward itself to know itself and mistakes its presence for existence (Higgins, 2013). This idea is based on a misunderstanding of the nature of nondual reflexivity. Non-dual awareness does not need to turn toward itself to know that it is aware. Its reflexivity is its essential property that makes it what it is; thus, it is spontaneously present and does not require an intentional transitive act of taking itself as an object in order to know itself (Gallagher and Zahavi, 2023; Josipovic, 2024).

The existence of nondual awareness is neither an inference, nor a belief, nor a sensation (Laukkonen et al., 2025). These are representational contents that may co-occur with nondual awareness, but they are not awareness itself. Non-dual awareness is said to exist-in-itself because, to itself, it is what is phenomenally ultimately real, as it is not constructed in dependence on concepts and other mental representations or conditioned by past experience (Rabjam, 2001, 2007; Josipovic, 2014, 2016, 2021, 2024). To nondual awareness, this is entirely obvious, or self-evident, as its being, as how it is, so it has no need to declare the status of its existence or non-existence. Because of this, it can also be said that it is empty of assertions of existence or non-existence (Lama, 2004; Rabjam, 2007).

## Reflexivity issues

Arguably, the second most significant obstacle to research is the belief that nondual awareness cannot know itself (Garfield, 2006, 2023, 2020; Kaul, 2024; Metzinger, 2024; Rabjam, 2007). It is based on misunderstanding the nature of nondual reflexivity by which this awareness knows that it is aware. As a result, what is most frequently researched are the related global states and phenomenal contents, rather than the extent to which nondual awareness is explicit in experience; in other words, the degree of its reflexivity (Josipovic, 2019, 2024).

Illusionist claims that the reflexivity of consciousness, in general, is merely a mistaken inference due to a failure of introspection have been refuted elsewhere and won't be restated here (Chalmers, 2020; Montague, 2016; Strawson, 2022; Zahavi, 2005). In respect to nondual awareness, the key argument can be summed up by saying that nondual reflexivity, by which consciousness itself knows that it is aware, is not a reflective introspection but an intrinsic property of this awareness. Unlike introspection and inference, which are conceptual processes that can be mistaken about nondual awareness, nondual awareness cannot be mistaken about itself, once its non-representational reflexivity is active (Josipovic, 2019, 2024).

The claim of mistaken inference is also based on the notion that consciousness is always a consciousness of something other than itself and, therefore, necessarily intentional and relational (Montague, 2016). This can be seen as being due in part to the unconscious semantic structuring of cognition into a subject and object and the misidentification of foundational nondual awareness with a conceptually reified subject who is attending to and monitoring contents and states (Josipovic, 2019, 2024).

A related objection is that nondual awareness cannot be inherently reflexive, but that it requires a secondary meta-cognition that is transitive and intentional, which takes this awareness as its object (Lama, 2004; Rabjam, 2007). Any knowing that needs to objectify awareness in this way is a conceptual knowing, no matter how subtle those concepts are. It is then knowing a conceptual representation of nondual awareness, and not this awareness itself (Josipovic, 2024). During meta-monitoring, in which the subjective aspect of experience seems absent, awareness can be present, but it appears as an object, as something other than the meditator (Dunne et al., 2019). This implies that there is still a subtle subject-object duality, of awareness as a subject knowing awareness as an object. Such transitive knowing is not how nondual awareness functions. Its presence is an indication that nondual awareness has not yet been realized (Josipovic, 2014, 2019, 2024).

Another widespread mistaken belief is that nondual awareness is altogether a state of “not knowing,” an undifferentiated existence in which there is no awareness (Mathes and Kemp, 2022; Radhakrishnan, 1994). However, phenomenally, the idea of existence without awareness is merely an idea within awareness, for without conscious awareness, nothing can be experienced. The issue here is that the usual conceptual processes that are relied on to know can be suspended and are absent in nondual awareness itself. This can then appear as an absence of knowing since nondual awareness does not rely on mental representations to know. Yet, it is knowing or being aware, just of a very different kind from what we are accustomed to. On the other hand, if a state of actual unconscious not knowing is experienced, it means that nondual awareness is still obscured by the substrate (Higgins, 2013; Josipovic, 2019, 2021, 2024; Mathes and Kemp, 2022). This can happen in cases when conceptual thoughts have been suspended, but the inherent nondual reflexivity has not yet been activated. A mistaken inference can then be made that nondual awareness is merely a cessation of thoughts (Rabjam, 2007).

A related belief is that nondual awareness cannot be present if there are any conceptual processes and thinking occurring simultaneously (Urgyen, 2004). This misunderstanding describes the early stages of acquaintance with nondual awareness, before abiding in and as awareness has been stabilized. It can also represent a commitment to a metaphysical belief that knowing via nondual awareness is a superior way of knowing and that all conceptual knowing should be eliminated (Rabjam, 2007). However, as long as nondual reflexivity, by which this awareness knows itself directly, has not been activated, nondual awareness remains as if it were some unknowable mystery, rather than simply a different way of knowing (Josipovic, 2019, 2025).

A variation of the above belief in “not knowing” is the belief that nondual awareness does not experience anything and that there is nothing it is like to discover it, and for this awareness to be explicitly present in experience. There are several different sources of this misunderstanding, aside from the obvious issue of the meaning of the term experience. One is the conflation of nondual awareness with a method used to attain it. Some methods aim to arrive at pure nondual awareness without any other content by developing absorption states in which other phenomenal content is diminished or entirely absent (Josipovic, 2019; Metzinger, 2024). With such methods, various states of absorption on the way to nondual awareness can be mistaken for nondual awareness itself. For example, one can enter states of deep mental silence without any other phenomenal content, but in which nondual awareness is still implicit or hidden, and then retrospectively infer that since nothing was experienced, this must have been a case of pure nondual awareness or pure consciousness, and that therefore there is no experiencing in nondual awareness (for detailed discussion, see Josipovic, 2019; Srinivasan, 2020). If, on the other hand, one has encountered awareness but is seeing it as a subtle object one is meditating on, as can occur in meditations on the nature of mind (Namgyal, 2006), then this is not yet nondual awareness, since nondual awareness does not contain even this very subtle duality.

A related belief is that for there to be any phenomenal experience or consciousness at all, there has to be a subject or self to whom that experience feels a specific way, depending on who that subject is (Zahavi, 2005). If the subject or self is absent, then there can't be anything it is like to experience consciousness or nondual awareness (Coseru, 2024; Fink, 2020; Schlicht, 2025). Regarding this, it is first important to understand that nondual awareness is not a specific experience that a subject has, the way one has an experience of seeing a movie or being in an altered state (Josipovic, 2019). Rather, it is that which is aware of any experience, whether naturally occurring or intentionally induced, whether intrinsically self-related or extrinsically other-related. Second, a constructed, conceptually reified self or subject is not necessary for experience because nondual awareness is itself that to which phenomenal content occurs (Josipovic, 2014, 2021). In other words, it is the one who is ultimately the conscious, aware presence in any experience. Therefore, nondual awareness is both without an experience and the most direct or real experiencing. Because it is without dualistic conceptual structures, it is neither an objective content or state that one is experiencing, nor is it a subject who is experiencing other contents and states as its objects (Josipovic, 2021, 2024, 2025). At the same time, it is the most direct conscious phenomenal experiencing that we can have, as it merely mirrors whatever content and state is present, without representing, re-representing, or editing them (Josipovic, 2025).

Another commonly held belief is that nondual awareness cannot know itself and know phenomena at the same time (Mingben, 2004). According to this belief, if nondual awareness knows itself, then it cannot know any phenomena that appear to it at the same time since it is occupied, so to speak, with knowing itself. Conversely, if it knows phenomena, it cannot at the same time know itself. Such beliefs are again based on a misunderstanding of the nature of nondual reflexivity, which, once explicitly activated, is non-transitive and constant, whether there are any phenomena present or not (Rabjam, 2007; Josipovic, 2019). Non-dual awareness merely mirrors any content and state co-occurring with it. Once its reflexivity has been activated, it cannot become unknowing of itself (Rabjam, 2001; Ksemaraja and Singh, 1990). Since the reflexivity of this awareness is its intrinsic property, rather than a result of some function, both its reflexivity and its mirroring of contents and states are a single nondual experiencing, nondual being. Therefore, mirroring phenomena does not require this awareness to abandon its reflexivity or to relate to phenomena as its intentional objects (Josipovic, 2024).

Finally, it is important to understand that there is a major qualitative cognitive shift between encountering various more or less unitary states of consciousness, including the formless lights and insights accompanying them, and when, eventually, the non-conceptual intrinsic reflexivity becomes activated and nondual awareness knows itself directly (Josipovic, 2019, 2021; Norbu, 2013; Rabjam, 2001; Wilkinson, 2018; Wallace, 2024). Once its reflexivity is clearly activated, nondual awareness cannot doubt that it is aware or that it is that which is aware in this direct, unmediated way. Thus, if subjects' confidence reports indicate that they are not certain whether they have encountered nondual awareness or if they are questioning how they can know that they have, it means that they have not yet realized it; that nondual awareness hasn't yet recognized itself. Instead, it is most likely still being mistaken for its reflections in meditation-generated states and contents, such as inner silence, peacefulness, positive affective states, relaxation of boundaries, etc. (Alcaraz-Sánchez, 2024; Hanley et al., 2025; Cardeña et al., 2025). Once this knowing has been clearly activated, nondual awareness knows itself directly as that which is aware. This non-transitive reflexive knowing is not an inferential belief based on reasoning about other beliefs. Thus, a requirement for epistemic justification does not quite apply here (Metzinger, 2024), as any justification of that sort is after-the-fact conceptual reasoning about the conceptual representation of nondual awareness, not its direct knowing of itself (Josipovic, 2024).

## Self-report and experiment design issues

Considerable progress has been made in recent years in dealing with various issues related to experiments on nondual awareness and related contemplative practices, including the development of new instruments and methods for self-reporting one's experience (Abdoun et al., 2024; Alcaraz-Sánchez, 2024; Czajko et al., 2024; Ehmann et al., 2025; Kok and Singer, 2017; Lutz et al., 2025; Medvedev et al., 2025; Metzinger, 2024; Milicevic et al., 2025; Sandved-Smith et al., 2025; Timmermann et al., 2025; Ventura et al., 2024; Zanesco et al., 2021).

Aside from the more general issues regarding self-reports (Van Dam et al., 2018), there are several issues related specifically to reporting nondual awareness. Reports of instances of isolated or pure nondual awareness (Metzinger, 2020, 2024) have raised questions about the possibility of reporting in the absence of memory and self (Fink, 2020; Schlicht, 2025). The issues of nondual awareness and self have been addressed above. The question about the absence of memory is based on not understanding that nondual awareness is always present in experience, whether isolated or not, and that it is orthogonal to working and long-term memory, and can therefore co-occur with them (Josipovic, 2021). Thus, while nondual awareness itself is not a reconstruction from memory, its presence does not interfere with memory functions. During explicitly manifest nondual awareness, there is no intentional maintenance and manipulation of conceptual-symbolic representations of nondual awareness in working memory, since it does not require semantic tagging in working memory to be present (for a more detailed discussion on this point, see Josipovic, 2019, 2021; also, Ricard and Singer, 2017; Laukkonen and Slagter, 2021; Metzinger, 2024).

Some of the same issues found in debates about phenomenal richness vs. sparseness (Block, 2023; Ji et al., 2024) may apply to reports on nondual awareness. How awareness appears phenomenally may seem ineffable (Rabjam, 1998). This is due to phenomenal richness that does not lend itself easily to being captured by concepts, which are its impoverished representations. Especially with nondual awareness, as the hierarchical layers of conceptual structures progressively relax and nondual awareness becomes less implicit, both the awareness and the phenomena co-present with it become more vivid, empty, luminous, and blissful (Hatchell, 2014; Josipovic, 2021; Rabjam, 2001; Wilkinson, 2018). Thus, not only nondual awareness, but any content or state present with it, may appear beyond words and descriptions. To a certain degree, one's lack of facility and practice in reporting on nondual awareness may be contributing to this ineffability. This would be akin to how those who grew up unaccustomed to noticing and expressing their emotions need time in therapy to learn to identify and talk about them. Conversely, one's previous understanding and beliefs about nondual awareness influence how one is experiencing and reporting it (Metzinger, 2024). In addition to the reverse possibility of phenomenal sparseness and cognitive richness (Gross and Flombaum, 2017), this issue is also related to the overall stages in one's acquaintance with nondual awareness, which, in general, progress from intellectual understanding, to insights, to experiences of altered states with nondual awareness as partially explicit, and, finally, to realization or activation of direct reflexivity when nondual awareness is fully explicit (Josipovic, 2021, 2024; Rabjam, 2007). Assessing where a participant is in terms of these four is non-trivial, as it does not necessarily scale with hours of contemplative practice. Terminology that participants are familiar with can influence how they report their experience. For example, some are more likely to report on perceptual content and leave out non-conceptual cognitive aspects, even in careful phenomenological or micro-phenomenological interviews (Petitmengine et al., 2019).

A potentially significant issue with self-reports is the *post-hoc* labeling of various states induced via contemplative practice or by mind-altering substances as nondual awareness. While nondual awareness is ubiquitous in experience, it is ordinarily only implicit, and it is no small feat for it to directly recognize itself without being mediated by concepts and other mental representations. This issue is especially important today, when the idea of awareness of awareness is gaining acceptance, and both participants and researchers are becoming all too eager to label this or that state as being nondual awareness (Josipovic, 2021, 2024).

A couple of speculative and rarely discussed issues concern how participants embody their experience or where in themselves they habitually live from, so to speak. These can influence how participants report on their experience of nondual awareness. This is related to the depth of interior contact with oneself (Blackstone, 2007, 2021). A shallow contact would be, for example, seeing the world and others from the surface of one's eyeballs and experiencing a certain degree of flatness, or feeling sensation mainly at the skin's surface without much interoceptive depth. These may correlate or be confounded with emotional flatness and shallowness of meaning. On the other end of that gradient, a deep contact would be experiencing the world and others from the center of one's core, with a corresponding depth of authentic emotion and meaning (for detailed discussion, see Blackstone, 2007, 2021).

A related issue is which aspect of their self the participants are predominantly identified with at the time of the experiment. For example, if predominantly identified as the one who controls or manages experience, there may be a tendency to report mainly in terms of meta-cognitive monitoring and optimization. If predominantly identified with the agentic self, there may be a tendency to report in terms of environment-related functions, like attention or connection. Or, if primarily identified with bodily homeostasis and allostasis, there may be a tendency to report mainly in terms of interoceptive sensation. Technically, none of these would be nondual awareness itself.

Finally, contemplative methods used to arrive at nondual awareness can be conflated with it and influence self-reports. For example, the strength and stability of focused attention or monitoring can be misreported as nondual awareness itself, especially during instances of reduced phenomenal content, either in absorption or in bare presence. Conflations with various pointing-out instructions are especially difficult to see through when nondual awareness is conflated with, among others, natural wakefulness, expanse of sky, the first moment of perception, nowness, resting in the present, ineffability, etc. (Metzinger, 2024). Mistaking such pointers for nondual awareness is akin to mistaking the proverbial finger pointing at the moon for the moon itself. The issue of conflating nondual awareness with various contemplative methods is rather profound and cannot be done justice here. Many volumes have been devoted to it over the centuries in various contemplative traditions (Namgyal, 2006; Rabjam, 2007). In terms of present-day research, it can be compared to how it has been easy to conflate consciousness with stimulus detection (Cogitate Consortium et al., 2025).

Perhaps the most challenging aspect of nondual awareness in terms of experimental research is its simultaneous transcendence and immanence. Researchers need to be clear on whether their target is nondual awareness as the transcendent, either isolated from other content as in pure awareness (Metzinger, 2020, 2024), or with phenomenal content as the aware epistemic space that encompasses and pervades content (Josipovic, 2014). Alternatively, their target could be nondual awareness in its immanent aspect as occurring within phenomenal content; in other words, its inseparability from content as that which gives it the phenomenal qualities that researchers are interested in (Boly et al., 2024; Czajko et al., 2024; Timmermann et al., 2023). Lastly, the target of research could also be the singularity of nondual awareness as the simultaneity of its transcendence and immanence (Josipovic, 2021). These choices should be made clear at the outset to avoid confusion in experiment design and in the interpretation of results.

Because it is distinct from and largely orthogonal to global state and local content, and to functions that influence them, nondual awareness can be present without any other phenomenal content, including any constructed self (Josipovic, 2019; Metzinger, 2024). It can then be mistaken for a mere absence of phenomenal content, a nothingness, as in an unconscious absorption (Cardeña et al., 2025; Josipovic, 2019; Srinivasan, 2020). On the other hand, because of its immanence, this awareness appears as nonduality or unity of all conscious contents and states (Josipovic, 2014, 2021). It can then be mistaken for the effect it has on content and state, or for some specific content or state (Josipovic, 2019, 2021, 2025).

In terms of specifying neural correlates of nondual awareness, its uniqueness means that these are most likely distinct from neural correlates of specific perceptual, affective, or cognitive contents, from the neural correlates of various functions such as attention, monitoring, memory, or meta-cognition, and from the neural processes involved in maintaining global states. Furthermore, such neural correlates would need to be able to function as the all-encompassing aware space within which different types of content, related to both the internal and external environment, self and other, could co-occur (Josipovic, 2014, 2021). When nondual awareness is explicit, its neural correlate would also need to be common across different states and contents. For example, if a neural signature of nondual awareness is not sufficiently the same when nondual awareness is isolated from other phenomenal content in absorption, as it is when it is present with full waking content, then this most likely would not be the signature of nondual awareness, but a signature of changes in the amount or type of content, or in the level of alertness and arousal (Josipovic, 2021).

Determining the specific neural correlate of nondual awareness is hampered by the general unruliness and complexity of the brain, its neural degeneracy, and its largely many-to-many organization, where each function can map to many brain areas, and each brain area can be involved in many functions by being recruited into different functional networks based on demand (He, 2023; Pesoa, 2018; Storm et al., 2024). These factors contribute to considerable intra- and inter-individual variability in neuroimaging data. Furthermore, as extraordinary as our current neuroimaging methods are, they still may be too crude to reliably detect the neural correlates of this most subtle aspect of human consciousness.

Broadly speaking, three types of neuroimaging experiments can be designed with the overall goal of specifying the neural correlates of nondual awareness. The first is aimed at differentiating the neural correlates of nondual awareness from those of different functions like focused attention, monitoring, working memory, metacognition, etc., from the neural correlates of interoceptive and exteroceptive perceptions, from different affective states occurring when nondual awareness is transitioning from implicit to explicit, and from natural and altered global states. The second is aimed at testing the intrinsic properties or dimensions of nondual awareness: its being, emptiness, luminosity, bliss, and unity, each of which occurs on the gradient from fully implicit to fully explicit. The third is aimed at assessing how perception, affect, and cognition change when nondual awareness is explicitly present, and how dimensions of nondual awareness are reflected in perceptual, affective, and cognitive contents.

From the many issues that such research would face, the following few are fairly obvious. Can the neural correlates of nondual awareness be differentiated from the neural correlates of residual content that may be co-present with it, such as blissful sensations, visual patterns, expansive and uplifting emotions, and various unitary states that entail loss of the self-other boundary, insights, etc.? For example, visual patterns appearing during meditation can occur while nondual awareness is in the implicit dualistic, transitional monistic, or explicit nondual zone of the gradient. Ordinary mental imagery is largely dualistic; visual effects of stabilizing attention on the way to pure awareness, such as kasinas in mindfulness practice or visions that are signs of energies dissolving into the central channel, are largely transitional; while the visions seen during Togel practice are, after some point, mainly nondual (Hatchell, 2014). Regardless of the type of visual content, or of how nondual they may appear, all these are phenomenal contents in awareness, not the nondual awareness itself. However, what exactly is the luminosity of nondual awareness neurally remains an open question. It has been reported that even when entirely isolated from other phenomenal content, nondual awareness appears to have an intrinsic luminosity to it (Fasching, 2021; Metzinger, 2024).

Contemplative methods practiced over many years can have rather loud neural signatures in the brain. Differentiating them from the neural correlates of nondual awareness can be challenging. For example, attending to bodily sensations will show as activations and correlations in areas of insulae and somatosensory cortex; monitoring practice in areas of the salience network; focused attention with or without an object in areas of the dorsal attention network; attending to self and self-related experience in areas of the default mode network; and, cultivating absorption states as a global cortical deactivation (for a more detailed discussion see Josipovic, 2021). Furthermore, altered states induced via psychedelics can have some similar effects on the brain as nondual awareness, such as increased criticality, but can also exhibit significant differences, such as increased within-network disorganization that is usually absent in nondual awareness (Carhart-Harris and Friston, 2019; Josipovic, 2014; Timmermann et al., 2025). Therefore, it may be better to test nondual awareness while minimally influencing the ordinary waking experience, especially since the technical description of such nondual awareness meditation is “resting in the nature of mind” (Josipovic, 2019, 2021; Koculak and Wierzchoń, 2022). This would mean testing how realized or self-recognized nondual awareness is; in other words, testing its reflexivity gradient (Josipovic, 2019, 2021, 2024). The issue then would be whether, based on the neuroimaging data, it could be reliably assessed whether nondual awareness is explicit, and whether its nondual non-representational reflexivity is active, or whether it is still implicit and obscured by conceptualizations. And if it is the latter, to what extent, and where it is on the reflexivity gradient (Josipovic, 2021, 2024).

Since nondual awareness is largely orthogonal to content and state, even though it may be implicitly or explicitly present in any experience, it does not by itself determine whether a specific content or state becomes available for conscious experiencing. For example, conscious seeing of visual stimuli, contrasted with trials of not seeing them, does not inform about nondual awareness, since nondual awareness is an unchanging aware space within which both the conscious seeing and the non-seeing occur (Josipovic, 2019, 2021; Czajko et al., 2024; for new developments see Fang et al., 2025). If such paradigms were to be used with participants who can reliably abide in reflexive nondual awareness, different classes of stimuli could be displayed, but the analysis would need to look not for differences but for those neural signatures that are common and remain relatively unchanged. This would not be without its own issues, as differentiating such signals from the unrelated and related physiological noise would not be trivial.
